# The Swedish Knee Arthroplasty Register (www.knee.se): The inside story

**DOI:** 10.3109/17453671003667267

**Published:** 2010-03-31

**Authors:** Kaj Knutson, Otto Robertsson

**Affiliations:** Department of Orthopedics, Lund University Hospital, SE-221 85 LundSweden

## Introduction

Professor Göran Bauer was the head of the Department of Orthopedics in Lund 1969–1989. The story goes that a failed surgery in the early 1970s, in which a left knee implant was inserted in the right knee (or vice versa), triggered his interest in monitoring the results of knee arthroplasty, which at the time was being performed with a variety of implants.

Bauer regarded knee arthroplasty as a large-scale human experiment and he thought it should be monitored. Who would benefit from these implants? Were they safe? What was the outcome? What were the types and rates of failure, and could they be managed? Thus, he became the major promoter of initiating a nationwide registration and in 1974, in collaboration with the Swedish Orthopedic Society, a meeting was held in Uppsala to decide on the matter. A group of about 20 interested surgeons attended and the majority voted for the project.

With monetary contributions from the Swedish Medical Research Council (MFR), the first national arthroplasty register was started in 1975 with its office located in Lund. Initially, not all operating units joined and although participation steadily grew, it was not until the early 1990s that the whole of Sweden was covered.

Professor Göran Bauer was responsible for the registry until his retirement in 1989, at which point Professor Lars Lidgren (who also attended the first meeting) took over. At the end of 2009, Lidgren stepped down but he will stay on as a patron and researcher.

## Financing

The MFR supported the registry for the first 5 years, after which there was a 6-year period with intermittent financing by a variety of research grants. In the mid-1980s, the Board of Health and Welfare (Socialstyrelsen) started to provide regular financial means but in 2007 the support of quality registrys was taken over by the Swedish Association of Local Authorities and Regions (SKL). In spite of this support by the central health authorities, the registry has been chronically under-financed, with Lund University Hospital indirectly bearing part of the cost and the rest being provided through individual research grants, e.g. from the Faculty of Medicine and Stiftelsen för bistånd åt rörelsehindrade i Skåne.

## Data gathering

Initially, the information was entered via a modem to a mainframe computer with limited power, with the data stored on tapes. Information about each procedure was coded to fill one printed line including identification number, hospital, diagnosis, date of surgery, side, type of surgery, implant type and brand, and early complications. In an attempt to evaluate the case mix, preoperative radiographs were studied and classified according to Ahlbäck at Saint Göran’s Hospital in Stockholm. The initial number of radiographs was manageable, but it grew steadily. Also, the quality of examinations varied considerably and made classification difficult; early on, this part of the study had to be terminated.

There was an ambitious follow-up program with an individual clinical follow-up form sent to each surgical unit at 1, 3, 6, and 10 years after surgery. These forms were used to validate previously recorded data and they also included a few outcome questions as well as surgeon and patient satisfaction. Thus, the registry used patient-reported outcome measures from the start.

Due to the increase in the number of operations, however, the workload of follow-up examinations was considered to be too laborious, resulting in a large number of incomplete forms. It was thus abandoned in 1989.

By this time, computers had become readily available and the registry had enlisted an orthopedic surgeon with computer skills, Stefan Lewold, who moved the data to an ordinary PC located at the office of the registry. Furthermore, it was decided to use decentralized computer reporting by units—which was groundbreaking at that time. As this was before the internet became widely available, Lewold designed a computer program for units to registry data both locally and on a diskette that could be sent to the registry. In the case of revisions, the registry was also provided with a copy of the medical charts from which the reason for and type of revision was classified. Thus, instead of ambitious data gathering, the registry had turned to a “minimal dataset”, as it was considered better to have complete information on few variables than incomplete information on many. Furthermore, this was believed to enhance the willingness of units to join the project and full national coverage was indeed achieved after this change in procedure.

In spite of the success of computer reporting, there were drawbacks. Firstly, there was no information on the exact part numbers of the components used and some surgeons kept on using obsolete generic names, which hampered analysis of implants. Secondly, it was unclear who had entered data (e.g. the surgeon or secretary) and from where the information had come (e.g. surgeon, discharge letter, or operation report). Thus, in 1999 the registry returned to paper-based reporting, basically using the same minimal dataset on a one-page A4 form, to which the stickers with the part numbers were added. Also, it was requested that the form should be completed in the operation theater where the implant stickers were accessible and where the information about the surgery would be as accurate as possible.

**Figure:**
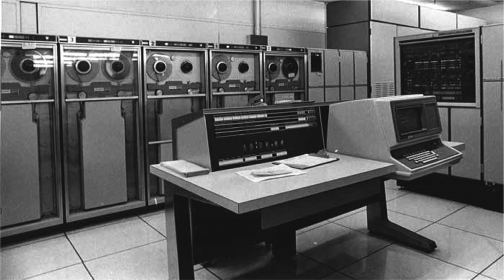
Initially a UNIVAC 1100/80 was used to process and store data. Today it is replaced by a laptop.

After having used this form successfully for 10 years, with full national coverage, it was felt that it would perhaps be possible to ask for additional data with reasonable compliance and without having a negative effect on participation. Thus, in 2009 a new one-page form was introduced that included questions about previous surgery on the affected knee, methods used (tourniquet, drainage, CAS, MIS), prophylaxis (infection, thrombosis), and also timing. Time will tell how successful this will be, but at this stage we are optimistic.

Apart from this regular gathering of data, the registry has acquired data for specific projects by sending questionnaires to patients. In this way, in 1997, 94% of all living registered arthroplasty patients responded to a questionnaire about possible unreported revisions and patient satisfaction. The registry also became one of the first registrys to use validated health questionnaires in 1998 when it evaluated what questionnaires were the most suitable for use after knee arthroplasty.

By the end of 2008, 150,000 primary knee arthroplasties and 10,000 revisions have been recorded.

## Analysis

Initially, the analyses performed by the registry were simple summary reports concerning the number of procedures with percentages of failures and revisions, as this was the customary way to report the results of surgery. However, a few years after the start, the registry began cooperation with the researchers at the Lund University Hospital Tumor Registry. There, the technique of calculating survival rate using actuarial life tables for cancer patients was available. This method for estimation of the survival of implants (using revision as an endpoint instead of death) was a novelty that became available to the registry. The first paper from the registry using this method was published in 1985 (Knutson et al.).

The improvement in life quality of arthroplasty patients was immense, and for the relatively few early complications it was difficult to know whether they were being completely and correctly reported. This, in combination with the lack of compliance with the extensive follow-up, forced the registry to focus on long-term outcome, measured using survival statistics with revision as endpoint. So it has been until the nineties, when the registry started to acquire data by mailing questionnaires to patients.

Since the start of using life tables, the statistical methods have evolved and become more sophisticated. In the late 1980s, Jonas Ranstam, a biostatistician, became a consultant for the registry. With his help, and later also with the help of his staff at the National Competence Center for Musculoskeletal Diseases (NKO), the registry has been able to stay at the cutting edge of medical statistics.

## Output

From the start, findings from the registry have mainly been presented at national and international meetings and published in peer-reviewed journals. An annual report was eventually made available, including some summary statistics to show those involved that the registry was alive and working. After the increase in the number of so-called quality registers and their common funding by health authorities, the annual report has become increasingly important—as it is in fact the only publication from the registry required by its financiers. However, at the registry we have been reluctant to use the annual report to publish fresh scientific findings. As the annual report is available worldwide on the internet, the likelihood of such findings appearing in peer-reviewed journals is reduced. We feel that the peer review is an important control of the quality of scientific research and that findings of importance should be scrutinized by external reviewers with respect to material selection, statistical methods, and the soundness of conclusions. The delay in publication by using scientific journals should not affect patient safety as any important information can, if needed, be provided to the hospitals directly or to the surgeons at the annual meetings of the registry.

## Mission

Initially, the registry was not intended to have any authority over the participating units and the choice of implant was left to the surgeon or his unit. Instead, it was hoped that the registry would able to identify any weaknesses in design, patient selection, or surgical methods early on—mainly because of the large number of cases involved—and thus help the profession to avoid unnecessary mistakes. The goal was also to help identify the underlying reasons for any deviations in results, and to be of assistance in the development of better implants and methods. This was to be achieved through the use of sound scientific methods.

In the late 1990s, however, the authorities discovered the potential benefits of registries and decided to join the bandwagon. The administrators wanted to maximize the benefit obtained from registries. From these new and old registries they wanted up-to-date information, delivered quickly in order to swiftly implement changes to reduce costs and improve both efficacy and quality.

This is of course a praiseworthy aim. The routines of some established registries such as the Swedish Knee Arthroplasty Register are not, however, adapted to acquiring short-term data. Given sufficient financing, this could of course be changed—as long as the new routines do not hamper the long-term objective of the registry. This may be a real risk, as it is known that there is some correlation between the amount of information requested and the likelihood of missing or incomplete reports.

Unfortunately, it seems that some administrators feel that studying data in order to improve quality does not need to fulfill the same standards as scientific research. The argument appears to be that public disclosure of results will stimulate those with seemingly inferior results to improve, irrespective of whether the results are afflicted by uncertainty or not. In addition, in an effort to achieve rapid results, there is a tendency for peer-reviewed publications to be replaced by administrative reports. With the stated goal of increasing the usefulness of the registries, the trend is that the authorities are taking over (from researchers) the role of dictating where the registers should be heading.

Our opinion is that quality control has to be up to the same standards as other research if it is to result in sustainable improvements, and that it is important that such activities should not eradicate the basic medical research for which the registers were intended.

We feel that historically, the registry has been at the cutting edge when it comes to scientific analysis of knee arthroplasty outcome. We hope that it will be able to stay that way in the future, while also managing to provide relevant information to its benefactors for the mutual benefit of patients and authorities.

Results from the Swedish Knee Arthroplasty Register have been included in several theses, articles, annual reports, and oral presentations. For a comprehensive list, see www.knee.se. A detailed description of the register was published in Acta Orthopaedica in 2000 (Robertsson et al.).
